# Probing Electrode Heterogeneity with 2D X‑ray Absorption Imaging

**DOI:** 10.1021/acs.jpcc.6c01717

**Published:** 2026-05-15

**Authors:** Min Li, Ralf Hendrik Menk, Fulvia Arfelli, Giorgia Bulli, Juan Reyes Herrera, Marco Giorgetti, Giovanni Agostini

**Affiliations:** † Elettra - Sincrotrone Trieste, s.s. 14, km 163.5, Trieste 34149, Italy; ‡ INFN, Section of Trieste, Trieste 34149, Italy; § Department of Computer and Electrical Engineering, Midsweden University, Sundsvall 85170, Sweden; ∥ Department of Physics, 243302 University of Trieste, Trieste 34127, Italy; ⊥ Department of Industrial Chemistry, University of Bologna, Campus Navile, Via Piero Gobetti 85, Bologna 40139, Italy; # School of Science and Technology, Chemistry Division, University of Camerino, Via Madonna delle Carceri, Camerino, Marche 62032, Italy

## Abstract

Unlike traditional X-ray absorption spectroscopy (XAS), two-dimensional (2D) X-ray absorption imaging, which combines spatial and spectral resolution, enables the spatially resolved analysis of compositional and chemical reaction heterogeneity within a material. An X-ray absorption imaging system with a large field of view (FoV) and high spatial resolution allows for the visualization and investigation of the structure–activity relationships in reactive systems. In this study, a 2D MiniPIX detector was installed at the XAFS beamline at Elettra to investigate the differing reaction mechanisms of manganese hexacyanoferrate (MnHCF) in two distinct electrolytes. By analyzing the spatially resolved X-ray absorption near-edge structure (XANES) data collected at the Mn k-edge, the concentration maps as well as phase evolution and distribution images of manganese species were obtained. These results further clarify the role of the Mn^2+^ additive in aqueous Zn-ion batteries and the working mechanism of MnHCF in such systems.

## Graphical abstract

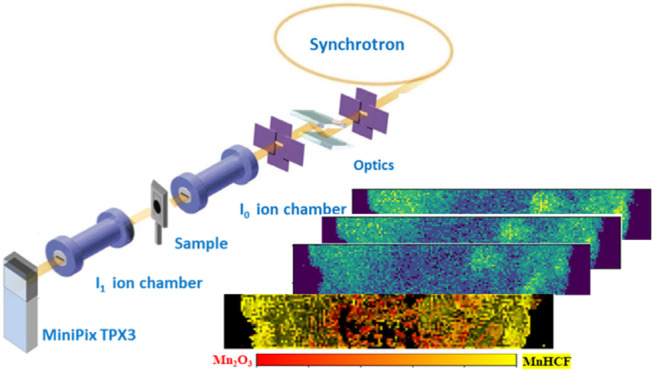

## Introduction

X-ray absorption spectroscopy (XAS) is a powerful element-specific technique for probing the local electronic and structural environment of a selected element within a material.^1–4^ Its working principle relies on scanning the incident X-ray energy across an absorption edge of a target element. By measuring the resulting absorption or emission, it provides averaged spectroscopic information from the entire illuminated spot. However, because the signal represents a statistical average over all probed atoms, conventional XAS cannot spatially locate or distinguish individual defects or unique local sites within a sample. This average limitation is particularly significant during *in situ* or *operando* experiments, such as the studies of catalytic reactions or battery cycling, where changes in the sample are often spatially heterogeneous.^5,6^ To address this, two-dimensional (2D) near-edge absorption structure (XANES) imaging offers a major analytical advantage. This technique directly correlates a material’s local chemistry with its spatial distribution, enabling researchers to visualize chemical heterogeneity, identify active sites, and observe phase transformations in complex systems.^7–11^

In battery research, a critical challenge is understanding capacity fading. It is widely accepted that chemical and material degradation processes, such as electrode dissolution or phase changes, electrolyte decomposition, and solid electrolyte interphase (SEI) instability, are responsible for the capacity loss and reduced lifetime.^12,13^ To elucidate the detailed mechanism behind this fading, 2D XANES measurements with high spatial and spectral resolution have been developed using synchrotron-based X-ray imaging techniques, such as scanning X-ray fluorescence (XRF) microscopy and full-field (FF) transmission X-ray microscopy (TXM).^5,14–20^ These methods provide invaluable insights into battery material evolution. In scanning XRF microscopy, a monochromatic X-ray beam is focused on a small spot and scanned across the sample point-by-point. By repeating scans over a range of incident energies near the absorption edge of a specific element, a 2D XRF-XANES dataset is assembled. This yields not only quantitative or semiquantitative elemental concentration maps but also information about the chemical state of the elements.^14,17^ However, due to its scanning nature, XRF microscopy generally requires a long acquisition time to achieve a reasonable signal-to-noise ratio for each pixel, which limits its suitability for studying large samples or dynamic processes. As an alternative, FF-TXM combined with XANES spectroscopy can provide 2D morphological and chemical-specific information on the scale of tens of nanometers. A single exposure on the order of 0.01 s is used to capture a snapshot of the entire absorption contrast image of the sample in the field of view (FoV), making it highly advantageous for dynamic, *operando* electrochemical studies.^18,19^ Nevertheless, FF-TXM has its own limitations: samples must be thin enough for X-ray transmission, and the image contrast is strongly influenced by the sample thickness and composition, particularly for light elements. Furthermore, high-resolution TXM typically operates with a very small FoV, constrained by the sensor and beam size, which makes it difficult to contextualize the high-resolution information within a large sample.

In this work, 2D XANES data of MnHCF Zn-ion battery electrodes were studied using a large, unfocused beam from a bending magnet source, by using a MiniPIX TPX3 detector (Advacam, Czech Republic, https://advacam.com/camera/minipix-tpx3/). The MiniPIX TPX3 is a miniaturized, low-power, single photon-counting imaging camera based on direct X-ray conversion. It employs the Timepix3 particle-tracking and imaging sensor, equipped with a 500 μm-thick silicon sensor bump-bonded to the Timepix3 readout application-specific integrated circuit (ASIC). The detector comprises 256 × 256 square pixels with a pitch of 55 × 55 μm, resulting in an active imaging area of approximately 14 × 14 mm. Each pixel can register up to 1022 photons per frame, and the maximum achievable frame rate is 16 frames per second using an USB 2 interface. For the measurements presented in this study, the energy threshold was set to 5 keV. Its high sensitivity and wide dynamic range enable the collection of high-quality XANES or EXAFS spectra within only a few minutes. For example, a XANES scan of the Mn k-edge from 6330 to 6730 eV, with a step size of 0.2 eV and an integration time of 0.2 s per point, requires a total acquisition time of 400 s. This capability is acceptable for capturing the real-time changes in chemical state distributions in *in situ* or *operando* reaction processes in our battery system.^21^ As previously reported,^17^ the MnHCF electrodes experienced significant compositional and structural changes during initial cycles in the aqueous Zn-containing electrolyte, due to the severe Mn dissolution and irreversible Zn^2+^ intercalation. Extended X-ray absorption fine structure (EXAFS) fitting results indicated the formation of a new MnO_*x*_ phase during cycling. However, when a Mn^2+^ additive is introduced into the ZnSO_4_ (3M) electrolyte, Mn dissolution is alleviated, and both XANES and X-ray diffraction (XRD) data show improved reversibility in the initial cycles.^22^ To visualize the differences of spatial composition and phase evolution in these two electrolytes, 2D XANES data were collected for the cycled MnHCF electrodes using this MiniPIX detector. Following data normalization and 2D XANES fitting, the concentration and phase distribution maps were obtained. These images spatially reveal a distinct reaction pathway in the two electrolyte systems. Such a spatially resolved chemical insight is essential for understanding structure–property relationships and for guiding the design of advanced functional materials.

## Experimental Section

The experiments were conducted at the XAFS beamline of Elettra Sincrotrone Trieste.^23^ As illustrated in Figure 1a,b, the setup followed a standard XAFS beamline configuration at a synchrotron radiation source, employing ionization chambers for transmission measurements. Additionally, the MiniPIX TPX3 detector was positioned downstream of the second ionization chamber (*I*_1_), allowing us to acquire reference data of the same sample with the standard ionization chamber setup. For the continuous scan, the current from the ionization chamber was measured using an AH501B 4-channel bipolar picoammeter, instead of the Keithley 428 current amplifier used for the step scan. The monochromator was continuously scanned across the K-edge of the element of Mn (6539 eV, from 6330 to 6730 eV with a step size of 0.2 eV, integration time: 0.2s) for the electrode samples.

**Figure 1:**
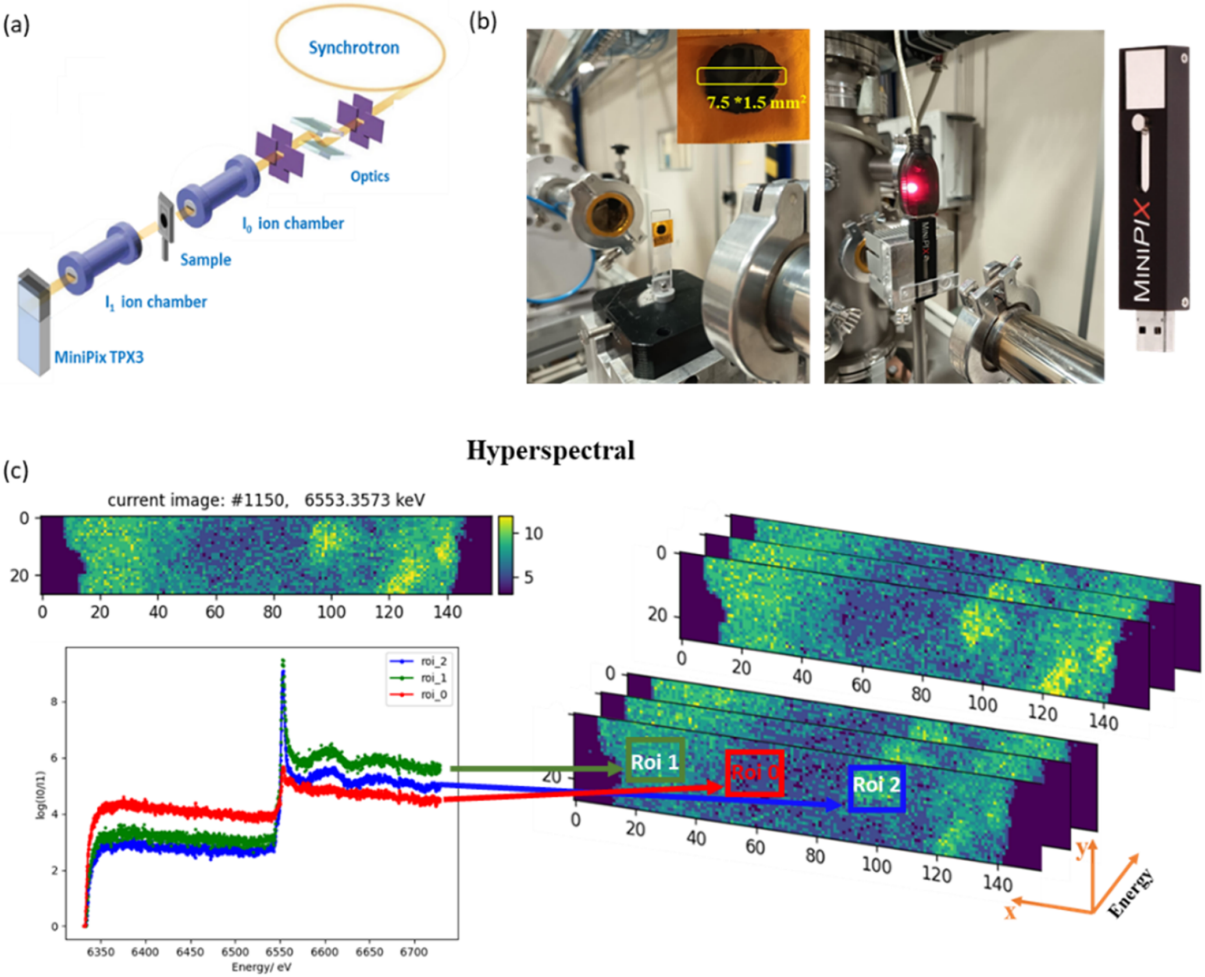
(a) Experimental setup installed at the XAFS beamline at Elettra Sincrotrone Trieste. (b) Photograph of the electrode sample and MiniPIX detector mounted at the XAFS beamline; the inset is the image of the sealed electrode with a beam size of 1.5 × 7.5 mm (h x w). (c) Depiction of some energy frames of raw data, as well as the mean value of the ROI intensities.

For all measurements discussed here, the MiniPIX detector was operated in frame mode, and 2000 frames were acquired during an edge scan covering a total energy range of 400 eV, with an integration time of 0.168 s per frame, corresponding to an energy step of approximately 1.0 eV/s. This integration time was selected to ensure sufficient statistical precision, considering that the measurements were performed at a bending magnet beamline. With the beam not focused, normalization was performed using two subsequent edge scans: one with the sample in place (*I*_s_) and one flat-field scan (*I*_f_) with the sample removed. Since the MiniPIX energy threshold was set to 5 keV, noise-triggered photon events extremely sparse and negligible even when compared to (cosmic) charged particle-induced traces; therefore, dark-field subtraction was unnecessary. Normalized images *N* (*x*, *y*, *E*_*i*_) were then generated pixel by pixel at the image position (*x*, *y*) for energy *E_i_* using the negative logarithm of the ratio

$$N\left(x,y,E_{i}\right)=-\log\left(\frac{I_{s}\left(x,y,E_{i}\right)}{I_{f}\left(x,y,E_{i}\right)}\right)$$

Along with the calibration and energy files, these data were input into Orange^24^ and PyXAS^25^ software for spectral calculation and fitting, followed by image segmentation.

The synthesis of MnHCF is based on a simple and reproducible coprecipitation method.^26^ The cathode was prepared by mixing 70% of the as-prepared MnHCF powder, 25% Super C65 (a high-purity carbon black conductive additive), and 5% polytetrafluoroethylene binder. The mixture was ground until a homogeneous thin solid slice was obtained. Subsequently, an 8 mm-diameter punch was used to form pellets with a mass loading of approximately 5–10 mg·cm^–2^. The electrochemical test was conducted using a 2032-coin cell, a small, round, flat primary battery with 20 mm diameter and 3.2 mm thickness: the active material was the working electrode and a zinc sheet (thickness 30 μm) was the reference and counter electrode, in 3 M ZnSO_4_ (Zn-only) and 3 M ZnSO_4_ + 0.1 M MnSO_4_ (ZnMn) aqueous electrolyte. The *ex situ* electrodes were collected using galvanostatic tests (current density: 20 mA g^–1^) with potential limitation in 1.0 < E < 1.9 V vs Zn^2+/^Zn. After the galvanostatic cycling, the cell was dissembled, and the cathodes were carefully extracted. The electrodes were then rinsed with flowing water, dried in an oven at 60 °C, and sealed with Kapton tape prior to XAS measurements. Figure 1c presents a frame of the cycled sample (C1 in Zn-only electrolyte) at a photon energy of 6553.3 eV, well above the Mn K-edge. The image area was defined by the size of the unfocused synchrotron beam, at the 1.5 mm × 7.5 mm (h × w) scale. The XANES graph in Figure 1c displays the energy-projected intensity files averaged over each ROI. The variations in the edge oscillations reflect the distinct chemical states of the Mn present in the sample, which we will discuss in the following part.

## Results and Discussion

Manganese hexacyanoferrate (MnHCF), as one of the Prussian blue analogues, has attracted considerable attention as a promising cathode material for post-lithium-ion batteries.^27–29^ However, in aqueous Zn-ion batteries, MnHCF suffers from severe manganese dissolution during electrochemical cycling. As shown in Figure 2a–c, the Mn distribution derived from the XANES edge jump was obtained for the pristine MnHCF electrode and the first charge (C1) electrodes cycled in Zn-only and ZnMn electrolytes. A clear decrease in the Mn content is evident in the cycled electrodes when viewed with a common color scale. Additionally, differences in the homogeneity of the Mn spatial distributions were observed. Compared to the pristine electrode, the C1 electrode cycled in a Zn-only electrolyte exhibited pronounced Mn depletion and increased heterogeneity. In contrast, the addition of Mn^2+^ to the electrolyte resulted in a relatively more homogeneous distribution of Mn.

**Figure 2:**
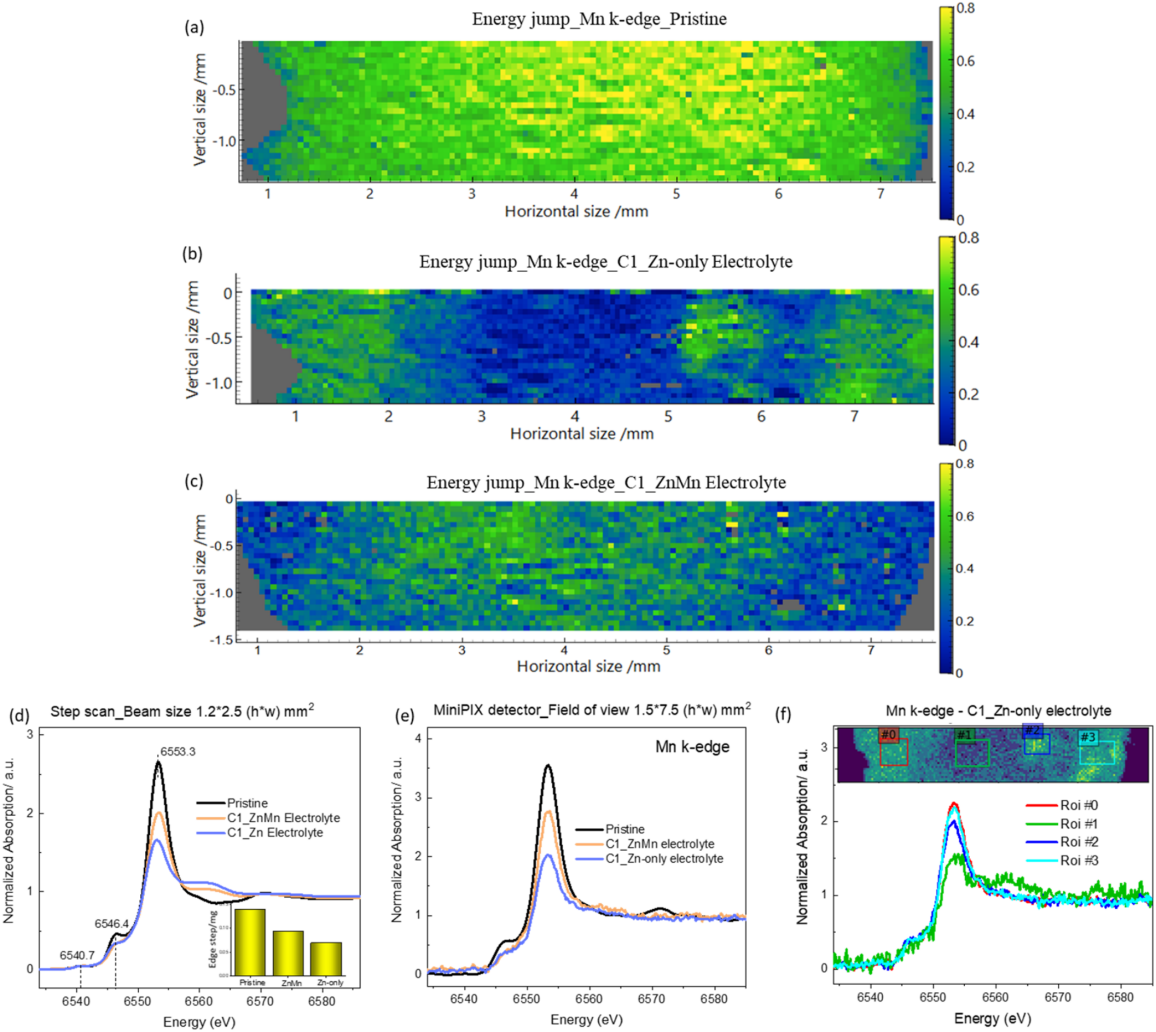
Mn distribution determined from the XAS edge jump measured at each pixel of the hyperspectral images for (a) pristine, first-charged (C1) electrode collected (b) in Zn-only and (c) in ZnMn electrolytes. (d) XANES spectra measured in step scan mode using an ionization chamber for the pristine and C1-cycled electrodes; the inset is the normalized edge step value got from Athena software. (e) XANES spectra of the electrode obtained in continuous scan mode from the MiniPIX detector by summing all pixels within the detector field of view. (f) Mn K-edge XANES data from the regions of interest (ROIs) in the Zn-only electrolyte.

For comparison, the XANES data of pristine and C1-cycled electrodes were measured using both an ionization chamber and the MiniPIX detector. As shown in Figure 2d, the XANES spectra acquired with the ion chamber exhibit high data quality, with a beam size of 1.2 × 2.5 mm (h × w). It is observed that the cycled electrodes show distinct differences in the pre-edge and rising edge (white line) regions compared with the pristine electrode. The inset shows that the edge jump value, which is proportional to the Mn content within the electrode, decreased for cycled electrodes. This is consistent with the images shown in Figure 2a–c. The XANES data collected with the MiniPIX detector are shown in Figure 2e. The displayed spectrum is an average extracted from all pixels and is consistent with the spectrum acquired by using the ionization chamber. However, slight differences are observed after the edge peak, which may be due to differences in the detection region and size. To correlate this spatial distribution of Mn with its corresponding chemical states, Mn K-edge XANES spectra were extracted from the selected regions of interest (ROIs), as shown in Figure 2f. Among them, ROI #1, characterized by a low Mn content, exhibits a noisy yet distinct XANES spectrum compared to other ROIs, potentially indicating a different Mn coordination or oxidation state.

A common observation from the XANES data collected using the ion chamber and MiniPIX detector is the decrease in the pre-edge peak and white line intensity. The pre-edge peak observed at around 6546.4 eV is attributed to the transition of the Mn *1s* electron to final states with significant Mn 4p character (hybridized 4p orbitals or Mn-to-ligand charge-transfer states).^30^ The intensity of this feature is strongly influenced by covalent mixing and local coordination geometry. The decrease in prepeak intensity observed for the cycled electrodes suggests either reduced covalent character in Mn–ligand bonds, increased local symmetry around the Mn center, a lower oxidation state, or a more ionic bonding environment.^31,32^ A decrease in the white line intensity at 6553.3 eV was also observed for the cycled electrodes. Since these C1 electrodes are charged electrodes, the Mn oxidation state is expected to increase, which would typically result in a higher density of unoccupied Mn 3d states and, consequently, a higher white line intensity. The observed decrease therefore points to structural reorganization during cycling, such as lattice distortion, collapse, or the formation of vacancies. These changes may involve the displacement of Mn from regular octahedral sites to distorted positions or a change of the coordination state from Mn–N_6_ (CN ligand) to mixed Mn–(N, O, vacancies) states.^33^ All of these reorganizations and associated ligand-field effects can modify the density of 4p or hybridized 4p-3d states, resulting in a lower white line intensity. This interpretation is consistent with our previously reported EXAFS fitting results, which confirmed the cleavage of the Mn–N bond with the formation of a Mn–O bond in an octahedral environment.^17^ Considering the different XANES spectra observed in ROI #1 (Figure 2f), obtaining distribution maps of different Mn components will enable a better understanding of the charging mechanisms of MnHCF in the two electrolyte systems.

To quantitatively map the distribution of specific manganese species resulting from electrochemical cycling, we employed linear combination fitting (LCF) to analyze the XANES data collected with the MiniPIX detector. Based on the previous EXAFS analysis,^17^ which confirms the formation of the Mn–O bond, MnHCF and Mn_2_O_3_ were selected as the two primary reference components for the LCF, following fitting trials with various Mn standards (MnO_2_, Mn_3_O_4_, MnSO_4_). This approach assumes that the spectra from each pixel can be represented as a weighted sum of these two known end-members. As shown in Figure 3a,f, the averaged fitted spectra from both electrolyte systems show excellent agreement with the measured data (R-factor <0.01). A high proportion of the Mn_2_O_3_ phase (0.34) was observed in the Zn-only electrolyte. In contrast, in the ZnMn electrolyte, the majority of Mn (0.92) remained in the MnHCF configuration.

**Figure 3:**
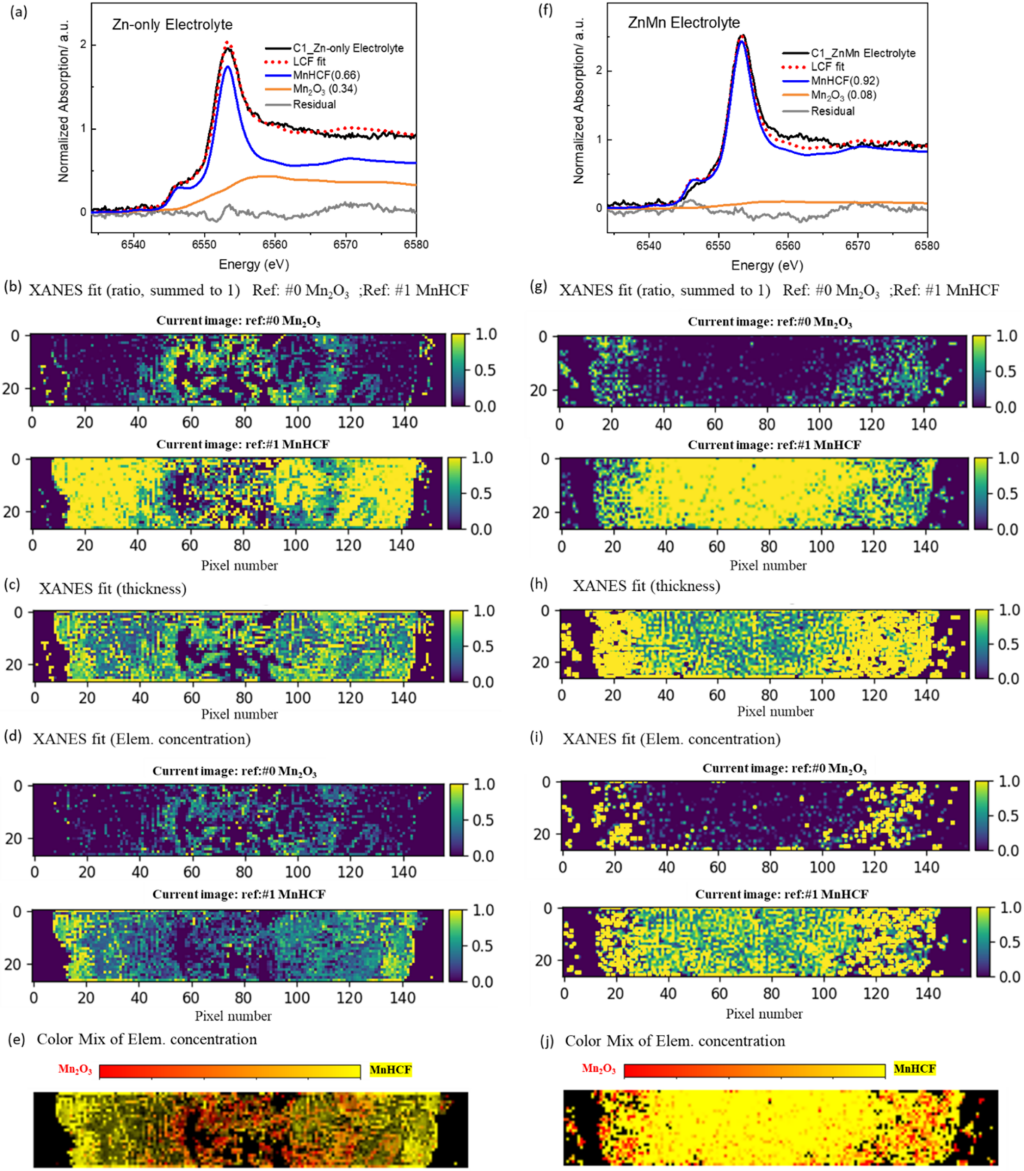
(a, f) Linear combination fitting of the XANES spectra from all the pixels for the C1 electrodes cycled in the Zn-only electrolyte and ZnMn electrolyte, respectively. (b, g) 2D distribution maps of the component ratios (Mn_2_O_3_ and MnHCF) in C1 electrodes cycled in the Zn-only electrolyte and ZnMn electrolyte. (c, h) Thickness image of the C1 electrodes cycled in Zn-only and ZnMn electrolytes. (d, i) 2D distribution maps of the component concentrations (Mn_2_O_3_ and MnHCF) for the C1 electrodes cycled in Zn-only and ZnMn electrolytes. Note: XANES fit (Elem, concentration) = XANES fit (ratio, summed to 1) x XANES fit thickness. (e, j) Color-mixed images of the concentration fitting, where red and yellow represent the distribution of Mn_2_O_3_ and MnHCF, respectively (pixel size: 55 x 55 μm).

Meanwhile, Mn_2_O_3_ and MnHCF were used as references for the 2D XANES fitting. The resulting distribution (ratio) of each phase is presented in Figure 3b,g. As observed, the distribution of the Mn_2_O_3_ phase differs between the two conditions. In the Zn-only electrolyte, the Mn_2_O_3_ phase is predominantly located in the middle part of the electrode, while in the ZnMn electrolyte, a high proportion of the Mn_2_O_3_ phase appears at the border. The ratio distribution of the MnHCF phase is relatively homogeneous in both electrolytes. As indicated in the fitting procedure,^25^ an “image thickness” is derived from the absorption edge normalization, which is approximately proportional to the local amount of the specific element (Figure 3c,h). Thus, the concentration map of each component is the product of the component ratio file and the thickness file (Figure 3d,i). It is observed that the Mn concentrations of both the Mn_2_O_3_ and MnHCF components are lower in the Zn-only electrolyte than in the ZnMn electrolyte, with a notably higher MnHCF concentration observed in the ZnMn electrolyte. The color-mixed images in Figure 3e,j clearly show the distribution of each component based on concentration. In both electrolytes, the distributions of the two components are observed to overlap considerably; however, the ratios and concentration of the components differ between the two electrolytes. This comparison clearly demonstrates the influence of the Mn^2+^ additive on the chemical state of Mn within the structure. By correlating these findings with the different electrochemical performances, the relationship between the chemical state of active metal and its electrochemical behavior is clearly established.

Since the component distribution image has been obtained, the detailed phase composition within specific ROIs can be readily determined through ROI-average fitting. As shown in Figure 4, the Mn_2_O_3_ phase is primarily concentrated in the central region of the electrode cycled in the Zn-only electrolyte. Consequently, two ROIs—one at the border (ROI: #0) and one in the central region (ROI: #1)—were selected for this electrode. An identical selection procedure was applied to the electrode cycled in the ZnMn electrolyte.

**Figure 4:**
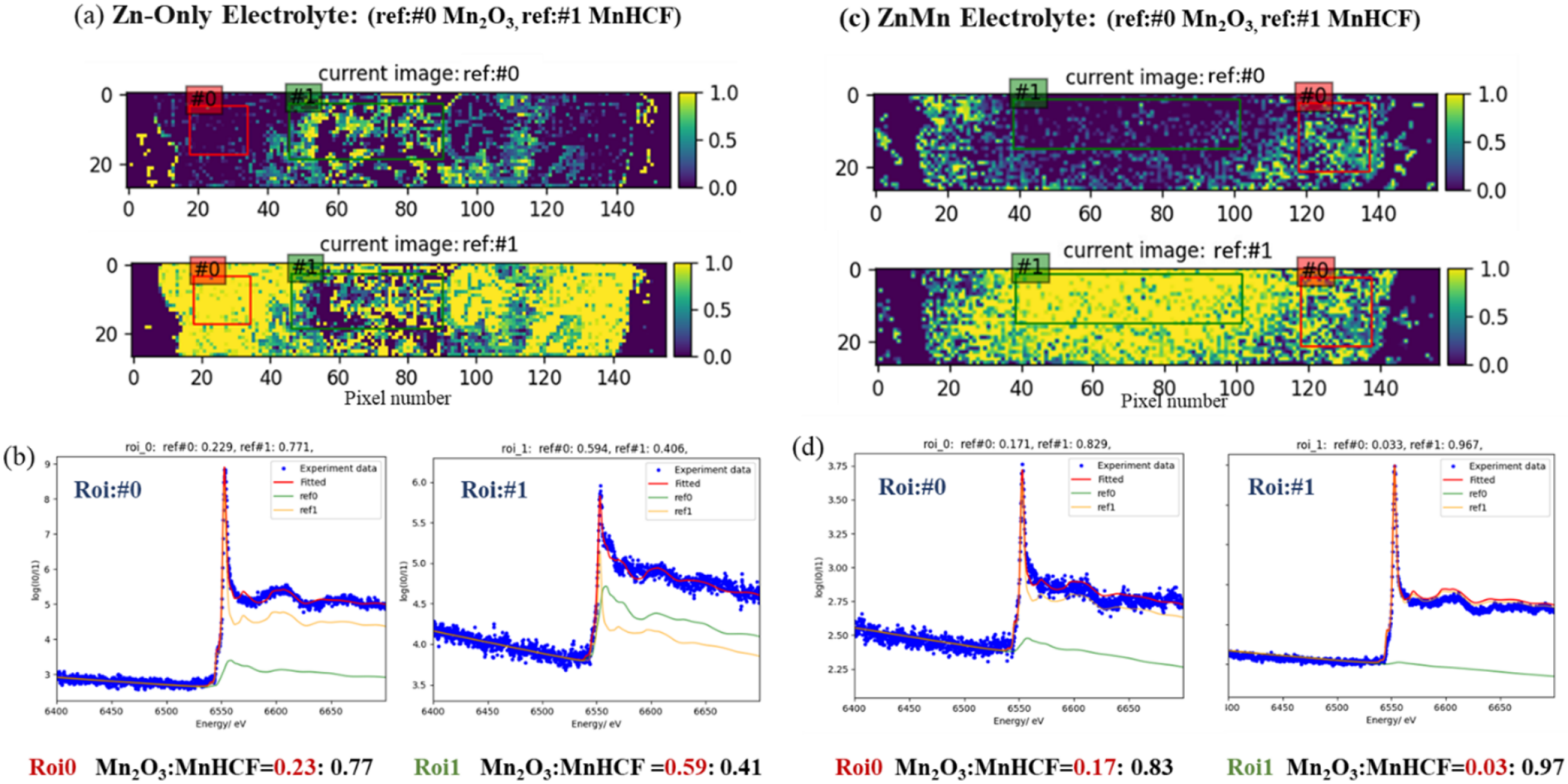
(a, c) ROI selection for C1 electrodes cycled in Zn-only and ZnMn electrolytes. (b, d) Corresponding fitting curves of the selected ROIs in the two electrolytes (pixel size: 55 × 55 μm).

For the electrode cycled in a Zn-only electrolyte, ROI #0 (border) shows a high proportion of MnHCF, with a MnHCF-to-Mn_2_O_3_ ratio of 0.77 to 0.23. In contrast, ROI #1 (center) exhibits a higher amount of Mn_2_O_3_, where the ratio shifts to 0.59 for MnHCF and 0.41 for Mn_2_O_3_. For the electrode cycled in the ZnMn electrolyte, the MnHCF phase is predominantly distributed in the central region. Quantification in ROI #1 (center) yields a MnHCF ratio of 0.97, which is higher than the average value obtained for the entire field of view (Figure 3f). Although the relative amount of the Mn_2_O_3_ phase is higher in the border region, its ratio is only 0.17 compared to the 0.83 for MnHCF.

In general, the concentration of the Mn_2_O_3_ phase is significantly lower in the ZnMn electrolyte than in the Zn-only electrolyte. This finding confirms the role of Mn^2+^ addition in suppressing the dissolution of MnHCF in an aqueous Zn^2+^ electrolyte and highlights the importance of a stable MnHCF structure for enhanced electrochemical performance. Unlike traditional XAS data, the 2D XAS approach, by combining spatially resolved imaging, enables direct visualization of the spatial distribution of specific element concentrations, as well as different oxidation states or chemical species, which is critical for understanding complex electrochemical processes. However, due to limitations of the coin cell transmission configuration used for aqueous Zn-ion batteries, particularly the thickness of the Zn sheet anode, all of the data in this study were collected *ex situ*. Future work will focus on optimizing both the cell setup and the MiniPIX detector to enable the *operando* analysis of the electrodes. As noted above, the readout of MiniPIX via USB2 imposes constraints on the achievable frame rate. This limitation can be mitigated, and buffered readout systems are available, which enable dead time-free data streaming. The MiniPIX, or more precisely the underlying Timepix3 ASIC, can operate at count rates of up to 10^6^ counts per pixel per second. For an integration time of 1 ms, this corresponds to a relative Poisson uncertainty of approximately 3% for a single-pixel signal. Maintaining this level of Poisson statistics effectively defines the intrinsic detector limit for operando battery studies.

## Conclusions

Two-dimensional XANES data were collected for cycled MnHCF electrodes using a MiniPIX TPX3 hybrid pixel detector. Following data normalization and 2D XANES fitting, concentration and phase distribution maps were obtained. These images not only reveal spatially distinct reaction pathways in the two electrolyte systems but also demonstrate the potential of this 2D-resolved X-ray spectroscopic imaging technique for tracking chemical and structural evolution in functional materials under *operando* or practical reaction conditions. With the advancements in synchrotron radiation sources and the continuous improvements in hybrid pixel detectors, particularly in spatial resolution, single-photon sensitivity, and energy discrimination, it will be possible to simultaneously acquire local XANES and EXAFS spectra across extended sample areas in the future. Such spatially resolved chemical insights are essential for understanding structure–property relationships and for guiding the design of advanced functional materials.
